# Humoral immunity to current variants of SARS-CoV-2 in exposed adults, September 2023 to September 2024

**DOI:** 10.1128/mbio.01618-25

**Published:** 2025-09-12

**Authors:** Lara M. Jeworowski, Barbara Mühlemann, Felix Walper, Marie L. Schmidt, Jenny Jansen, Andi Krumbholz, Terry C. Jones, Victor M. Corman, Christian Drosten

**Affiliations:** 1 Institute of Virology, Charité - Universitätsmedizin Berlin, corporate member of Freie Universität Berlin, Humboldt-Universität zu Berlin, and Berlin Institute of Health 9373, Berlin, Germany; 2 Faculty of Life Sciences, Humboldt Universität zu Berlin 9373, Berlin, Germany; 3 German Centre for Infection Research (Deutsches Zentrum für Infektionsforschung) 459706, Berlin, Germany; 4 Institute of Medical Microbiology, Christian-Albrechts-Universität zu Kiel and University Medical Center Schleswig-Holstein, Campus Kiel 9179, Kiel, Germany; 5 Laboratory Dr. Krause und Kollegen MVZ GmbH, Kiel, Germany; 6 Centre for Pathogen Evolution, Department of Zoology, University of Cambridge 98530, Cambridge, United Kingdom; 7 Labor Berlin–Charité Vivantes GmbH 646268 , Berlin, Germany; McMaster University, Hamilton, Ontario, Canada

**Keywords:** SARS-CoV-2, humoral immunity, immune escape, population immunity

## Abstract

SARS-CoV-2 evolves variants that evade population immunity. Monitoring group-level immunity is critical for assessing population susceptibility to newly circulating variants and updateability of imprinted immunity after exposure. We established a closely monitored cohort of 58 fully vaccinated adults in Berlin, Germany. Of these, 49 had at least one previous Omicron infection. In September 2023 and again in September 2024, we analyzed neutralizing antibody responses using full-virus plaque reduction neutralization tests against seven SARS-CoV-2 variants: B.1, BA.2, BA.5, EG.5.1, JN.1, KP.3.1.1, and XEC. Vaccination and exposure histories were traced using medical records, RT-PCR testing of any episode of respiratory tract infection, and serological testing for subclinical infections. Infecting variants were determined by sequencing or from unequivocal variant circulation at the time of positive testing. Titers from September 2023 included responses to both then-current and future variants. Over the study period, 13 subjects received monovalent XBB.1.5 vaccine. Thirty-four had one, and five more than one SARS-CoV-2 infection. None of the subjects was exposed to the most recent variant, XEC. Neutralization titers against all tested variants increased over time. Highest fold increases were seen against KP.3.1.1 and XEC. Reactivity profiles differed by exposure histories reflecting the most recent variant contact. Exposure to new variants leads to relative updates in population-level neutralizing antibody activity. Despite these updates, absolute group-level neutralization activity was low in September 2024 due to low titer levels against currently circulating variants KP.3.1.1 and XEC. Ongoing monitoring is needed to assess the need for further vaccine updates.

**IMPORTANCE:**

As new Severe Acute Respiratory Syndrome Coronavirus 2 (SARS-CoV-2) variants continue to emerge, understanding how population immunity evolves is essential to guide vaccine updates and public health strategies. Our study follows a group of fully vaccinated adults for 1 year (September 2023 to September 2024) to track how infection and vaccination affect the ability to neutralize new viral variants. Despite the continued emergence of immune escape variants, the results show that infection with recent variants helps to “update” immunity at the group level, even against newer variants such as KP.3.1.1 and XEC, although titers to new variants were low, confirming the existence of immune imprinting. These findings suggest that exposure to new variants adapts the immune system over time. This provides valuable insight into how populations build resilience against SARS-CoV-2 and whether updated vaccines are needed.

## INTRODUCTION

The fourth year since the onset of the SARS-CoV-2 pandemic continued to see the ongoing evolution of SARS-CoV-2 variants (https://www.who.int/activities/tracking-SARS-CoV-2-variants/). In mid to late 2023, the BA.2.86 variant and its descendant JN.1 were first reported in multiple countries (https://www.who.int/docs/default-source/coronaviruse/21112023_ba.2.86_ire.pdf, https://www.who.int/docs/default-source/coronaviruse/18122023_jn.1_ire_clean.pdf?sfvrsn=6103754a_3). Compared to their ancestral variant (BA.2), variants BA.2.86 and JN.1 had 32 and 33 amino acid substitutions in the spike protein, respectively, and JN.1 rapidly became the globally dominating variant in January 2024 (https://gisaid.org/hcov-19-variants-dashboard/). In early 2024, three JN.1 descendant variants JN.1, KP.2, KP.3, and its descendant KP.3.1.1, arose, with KP.3.1.1 dominating circulation worldwide by September 2024 (https://cov-spectrum.org/explore/World/AllSamples/from%3D2024-06-01%26to%3D2025-04-08/variants?nextcladePangoLineage=KP.3.1.1*&). All have three to four amino acid substitutions in the spike protein compared to JN.1 and were classified as variants under monitoring by the WHO (https://www.who.int/activities/tracking-SARS-CoV-2-variants/). In June 2024, the recombinant XEC variant arose and co-circulated during the second half of 2024 at high levels with KP.3.1.1 (https://www.who.int/docs/default-source/coronaviruse/09122024_xec_ire.pdf). XEC is a recombinant of KS.1.1 and KP.3.3 and differs from KP.3.1.1 at three positions in the spike protein (1).

The ongoing evolution of immune escape variants has been countered by recurring vaccine updates. Since April 2024, the WHO has recommended the use of JN.1 lineage vaccines, including monovalent JN.1, KP.2, and most recently LP.8.1 vaccines (https://www.who.int/news/item/26-04-2024-statement-on-the-antigen-composition-of-covid-19-vaccines, https://www.who.int/news/item/15-05-2025-statement-on-the-antigen-composition-of-covid-19-vaccines). It is currently unclear whether the future trajectory of population immunity will require further vaccine updates. Whereas mounting population-level cellular immunity may confer additional protection, it remains a subject of debate whether and to what extent re-exposure to current variants confers an update toward group-level immunity that can overcome the imprinting on B-cell immunity by past variants. One major challenge in answering this question is the scarcity of relevant SARS-CoV-2 data following the discontinuation of testing and tracing programs.

To provide insight into the development of group-level immunity upon re-exposure with current variants, we assembled a well-characterized longitudinal cohort of adults living in Berlin (2). We followed the development of group-level neutralizing immunity from 2023 to 2024 with steady monitoring of respiratory tract infection (RTI) episodes and virus neutralization testing against recently circulating variants. While we observe clear neutralization escape by recent variants, there is also a clear indication of an updating of group-level immunity due to natural exposure to recent variants.

## RESULTS

### Cohort

To assess changes in neutralization activity against emerging SARS-CoV-2 Omicron variants in a cohort with known exposure histories, we collected sera from 58 individuals in September 2023 and re-sampled the same individuals 1 year later. The individuals represented a predominantly young and healthy population resident in Berlin and surrounding areas (median age 35 years, age range 22–60 years, 57% female) (Table 1). By September 2023, all individuals had received at least three vaccinations against the SARS-CoV-2 wild-type variant using mRNA (Comirnaty, Pfizer, New York, the United States/BioNTech, Mainz, Germany or Spikevax, Moderna Biotech, Madrid, Spain) or vector-based (Vaxzevria, AstraZeneca, Cambridge, the United Kingdom) vaccines and 49 had at least one infection identified by PCR, rapid antigen tests, and/or detection of anti-SARS-CoV-2 nucleocapsid antibodies. All reported infections had taken place during the time of circulation of the Omicron variants. Between September 2023 and September 2024, 13 individuals received a monovalent booster vaccination against XBB.1.5 (Comirnaty, Pfizer, New York, the United States/BioNTech, Mainz, Germany) and 34 had at least one and 5 at least two SARS-CoV-2 infections, as confirmed by PCR or rapid antigen test or identified by increase of titers against its nucleocapsid protein. When sera were taken in September 2023, the EG.5.1 variant had dominated circulation during the preceding months in Germany. Between our two sampling time points, BA.2.86 and JN.1 dominated circulation in Germany until April, when circulation of JN.1 descendants, mainly KP.3 and KP.3.1.1, increased rapidly (https://public.data.rki.de/t/public/views/IGS_Dashboard/DashboardVariants?%3Aembed=y&%3AisGuestRedirectFromVizportal=y). XEC was first detected at the end of June (https://public.data.rki.de/t/public/views/IGS_Dashboard/DashboardVariants?%3Aembed=y&%3AisGuestRedirectFromVizportal=y). We assume no XEC contact occurred in our cohort, as infections were either sequenced or infections took place in July or earlier when XEC was barely detected.

**TABLE 1:** Demographic and clinical cohort characteristics

	2023	2024					
	Total	Total	No exposure	Unassigned exposure	EG.5.1 exposure	JN.1 exposure	KP.3 exposure
Number of individuals	58	58	17	6	15	11	9
Age in years (median, range)	35, 22–60	36, 23–61	37, 25–61	35.5, 23–48	40, 28–48	31, 26–61	38, 29–45
Sex							
Female	33	33	6	3	10	8	6
Male	25	25	11	3	5	3	3
Number of COVID-19 vaccinations							
3	36	34	10	4	8	6	6
4	20	12	7	1	2	2	0
5	2	11	0	1	4	3	3
6	0	1	0	0	1	0	0
Number of SARS-CoV-2 infections							
0	9	0	0	0	0	0	0
1	34	16	7	1	6	1	1
2	15	34	10	3	8	8	5
3	0	8	0	2	1	2	3
Antigen contact 3 months prior to blood draw							
No	51	45	17	2	15	11	0
Yes	7	13	0	4	0	0	9

### Serum neutralization in September 2024 compared to September 2023

**Fig 1:**
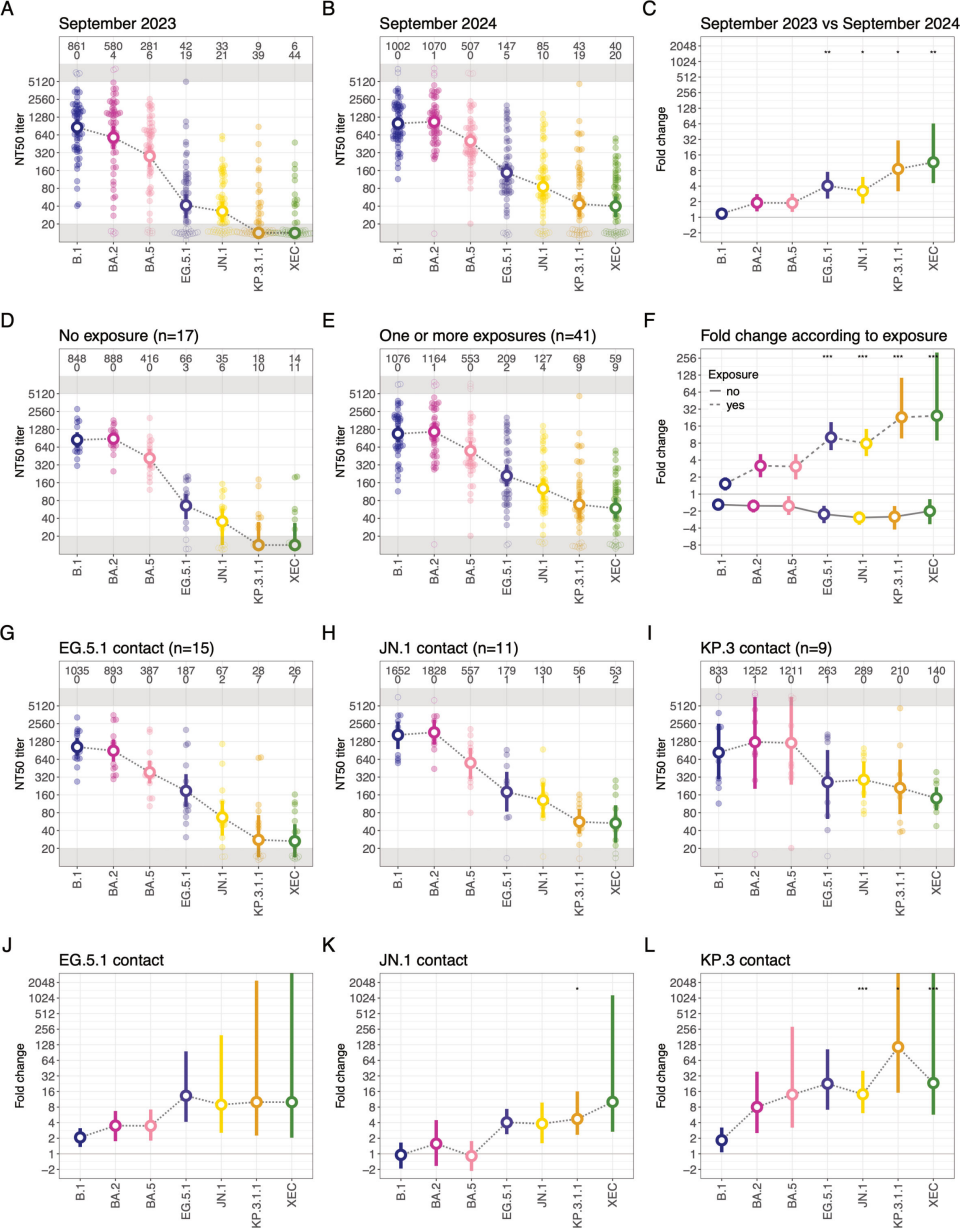
Titer magnitudes and fold changes. (**A**) Titers of 58 individuals sampled in September 2023. (**B**) Titers of 58 individuals sampled in September 2024. (**C**) Fold difference between titers of 58 individuals sampled in 2023 and 2024. (**D**) Titers of 17 individuals sampled in 2024 without exposure to SARS-CoV-2. (**E**) Titers of 41 individuals sampled in 2024 with one or more exposures to SARS-CoV-2. (**F**) Fold change of individuals with (dashed line) and without (solid line) exposure to SARS-CoV-2 compared to their titers in September 2023. (**G–I**) Titers of individuals sampled in September 2024 with exposure to (**G**) EG.5.1 (15 individuals), (**H**) JN.1 (11 individuals), and (**I**) KP.3 (9 individuals). (**J–L**) Fold change of individuals with exposure to (**J**) EG.5.1, (**K**) JN.1, or (**L**) KP.3 compared to their titers in September 2023. In (**A, B, D, E, G–I**), small dots show individual titers, large dots and ranges the Geometric mean titers (GMTs) and 95% highest posterior density (HPD) interval. Titers and GMTs falling below the detection threshold of 20 are plotted in the gray region at the bottom of the plot, titers >5,120 are plotted in the gray region at the top of the plot, with vertical jitter added. Numbers at the top indicate the GMT (top row) and the number of individuals with non-detectable titers (bottom row). In (**C, F, J–L**), dots and ranges show the mean and 95% HPD interval of the fold change. Stars denote *P*-values (empty: not significant, *P < 0.05, **P < 0.01, ***P < 0.001). *P*-values were calculated using a two-sided Student’s *t*-test and adjusted using the Bonferroni correction.

Neutralization titers of all sera were measured by PRNTs (Fig. 1A through C). In 2023 sera, titers were highest against the wild-type B.1 followed by BA.2 and BA.5 variants. Titers were substantially lower against concurrent EG.5.1 and JN.1 and future KP.3.1.1 and XEC. Of 58 individuals, 19 and 14 had detectable titers against KP.3.1.1 and XEC, respectively, even though these variants had not circulated in Germany when the sera were taken. In 2024, titer magnitude against B.1 was similar, while titers against other variants were generally higher than in 2023, with a broadly similar reactivity profile. An increased number of individuals had detectable titers against KP.3.1.1 and XEC (39 and 38, respectively). The largest fold increase in titers between 2023 and 2024 was observed for XEC, KP.3.1.1, and EG.5.1.

### Changes of serum neutralization depending on exposure

Of the 58 individuals included in this study, 41 were exposed to SARS-CoV-2 antigens at least once between September 2023 and September 2024, through vaccination (*n* = 2), infection (*n* = 28), or both (*n* = 11). In the remaining 17 individuals, no exposure was reported or detected by an increase of anti-SARS-CoV-2 nucleocapsid antibody titers (Table 1). When analyzing the individuals based on exposure during the study period, we find similar or slightly decreased titers in September 2024 compared to September 2023 for the individuals without an exposure (Fig. 1D and E), in line with waning antibodies over time. We find increased titers for the individuals that were exposed to SARS-CoV-2, with titers increasing the most for KP.3.1.1 and XEC, and only a limited increase in titers for B.1 (Fig. 1E and F).

### Impact of the variant exposure on serum neutralization

To determine the impact of the variant an individual was exposed to, we split our 2024 cohort according to the most recent variant each person had encountered (Fig. 1G through L). Fifteen individuals were exposed to the XBB/EG.5.1 group of antigenically related viruses (referred to as EG.5.1 contact group), 11 to BA.2.86 and JN.1 sublineages (JN.1 contact group), and 9 to KP.3 and its descendants (KP.3 contact group) (Table 1). None of the individuals had contact with XEC. We found that where the most recent antigen contact was with the EG.5.1 variant, titers against EG.5.1, JN.1, KP.3.1.1, and XEC were boosted similarly, with lesser titer increase against BA.5, BA.2, and B.1 (Fig. 1J). The individuals with JN.1 as the most recent contact showed similar fold change against the EG.5.1, JN.1, KP.3.1.1, and XEC variants, and no evidence for an increase in titers for B.1, BA.2, and BA.5 (Fig. 1K). In contrast, in the KP.3 contact group, the highest fold increase was for the homologous KP.3.1.1 variant (Fig. 1L). Reactivity against BA.2, BA.5, JN.1, and XEC also increased, while reactivity against B.1 did not. Titers and fold increase in the KP.3 contact group was highest, consistent with these individuals having been exposed most recently.

## DISCUSSION

Monitoring group-level neutralizing antibody activity against SARS-CoV-2 is important for assessing population susceptibility and the need for vaccine updates. Previous vaccine or virus exposure may change reactivity to certain substitutions and hence affect individual susceptibility to recent virus variants (3). The extent to which immunological imprinting hampers the ability of neutralizing antibodies to adapt to recent variants is currently under debate. For instance, a recent study found XBB.1.5 monovalent vaccine elicited predominantly cross-reactive antibodies on the basis of previous B.1-type vaccination, as opposed to XBB.1.5-specific antibodies (4). However, others found that a subgroup of subjects developed XBB.1.5-specific neutralizing antibodies in a similar setting (5).

Many studies stratify cohorts according to vaccine exposure, often discriminating between groups with or without booster vaccinations subsequent to first course immunization, and often recording concomitant natural exposures without reference to variant. We focused on a cohort with age- and time-typical vaccination history that is precisely stratified for natural infection with a relevant panel of most recent viruses. Focusing on the most recent evolution of immunity during September 2023 to September 2024, we found increased group-level neutralization against all variants and an overall highest fold increase against the most recent variants KP.3.1.1 and XEC. Since none of the individuals were exposed to the then upcoming variant XEC, and only nine were exposed to concurrent KP.3.1.1, this indicates the induction of cross-neutralizing antibodies to descendant variants by exposure to immediate precursors of these variants. This is in accordance with recent findings showing that existing B memory cells elicited by older vaccines can adjust their specificity toward newly evolving variants, as shown by the example of Omicron BA.5 exposure that improved group-level immunity toward subsequent HK.3 and JN.1 strains (6). Another study found that natural exposure with recent Omicron strains, particularly JN.1, in multiply-vaccinated subjects induced a broadened response to all variants up to JN.1 (subsequent variants were not assessed) (7). Despite the clear existence of immunological imprinting in these studies and ours, the collective results suggest an immunity-updating effect conferred by recent natural exposure to circulating viruses, affecting these viruses and their descendants.

Generally low titers and high fold increase against KP.3.1.1 and XEC variants indicates an antigenic difference of these two variants compared to previously circulating variants, though we find no evidence of additional escape of XEC compared to KP.3.1.1. This is consistent with other studies assessing neutralizing antibodies in sera from XBB.1.5 and JN.1 exposed individuals using full virus (8) or pseudovirus neutralization assays (1, 9–11). In contrast, others have shown a slightly stronger immune evasion of XEC compared to KP.3.1.1 in individuals with JN.1 exposure (12, 13). In sera from KP.3.1.1 and KP.3.3 breakthrough infections, two studies describe a slightly enhanced immune escape of XEC compared to KP.3.1.1 (1, 12). In our KP.3 exposure sub-group, we also see slightly reduced titers against XEC compared to KP.3.1.1, highlighting the impact of the infecting variant on the reactivity profile of sera. When analyzing serum reactivity according to the variant of exposure, we found that sera from individuals exposed to JN.1 had on average lower titer increase, in particular against B.1, BA.2, and BA.5 compared to individuals exposed to EG.5.1 and KP.3.1.1. Broader reactivity after EG.5.1 compared to JN.1 infection is also supported by hamster sera (14). However, higher reactivity may also reflect more recent antigen contacts, as possibly indicated by the relatively higher reactivity after KP.3.1.1 infection.

Our study has several limitations. The overall age composition might not quantitatively reflect older populations with underlying disease and a lesser degree of immunological adaptability to novel variants. Also, while neutralizing antibody activity is considered a valid surrogate of variant-specific immunity, cellular immune functions are not affected by viral antigenic drift to the same degree and their contribution to group-level immunity is not considered here. While evaluation of neutralization at specific timepoints provides valuable insights into population immunity, it is less informative regarding the impact of the exposure to specific variants, as effects might be masked by waning of antibodies over time. The small cohort size and the considerable number of individuals with multiple variant contact did not allow us to evaluate single contact categories. In addition, the occurrence of unrecognized infections detected by increases of anti-SARS-CoV-2 nucleocapsid antibody levels and the lack of sequencing data for some of the reported infections restricted the exposure classification and might have led to misclassification. Despite the limitations, our results provide a comprehensive overview of the evolution of neutralization titers in a well-characterized adult cohort in Berlin, Germany.

## MATERIALS AND METHODS

### Subjects and samples

Serum samples were collected from the same 58 subjects in September/October 2023 (09/08–10/13) and August/September 2024 (08/29–09/26). SARS-CoV-2 vaccination histories and anamnestic histories of any SARS-CoV-2 infection in the preceding year were recorded upon both visits. Each subject additionally submitted self-sampled respiratory swab specimens for broad-range (RT-)PCR testing during every RTI episode in the observation period (September 2023 to September 2024). To identify asymptomatic or subclinical virus infections, antibody levels against the SARS-CoV-2 nucleocapsid protein were monitored every 3 months in every subject during the observation period using the Elecsys anti-SARS-CoV-2 test (Roche, Mannheim, Germany) (Table S1).

The variant an individual was infected with was assigned based on the genome sequence recovered from nasopharyngeal swabs in voluntary testing, if available, or according to predominance of the respective variant at the time of infection in cases of subclinical exposure. In times of codominance of multiple variants, no variant assignment was made.

Exposures (vaccinations and infections) were categorized according to antigenic similarity as seen in the clustering of variants in SARS-CoV-2 antigenic maps (15, 16): the EG.5.1 exposure group includes contact with XBB and EG.5.1 sublineages, the JN.1 exposure group includes BA.2.86 and JN.1 sublineage contact, and the KP.3 exposure group includes KP.3 descendants like KP.3.1.1 but not XEC. Individuals were classified according to their most recent antigen contact. Hence, some individuals of the JN.1 and KP.3 contact groups had an additional recorded exposure to earlier variants (EG.5.1 or JN.1, respectively).

Prior to testing, serum samples were heat-inactivated at 56°C for 30 min.

### Cell culture and viruses

Vero E6 cells expressing the transmembrane serine protease TMPRSS2 (Vero E6/TMPRSS2, NIBSC 100978) were grown in Dulbecco’s modified Eagle’s medium (DMEM, Gibco, Darmstadt, Germany) supplemented with 10% fetal calf serum (Gibco) and 1.0 mg/mL geneticin (G418, Gibco). The cells were incubated at 37°C in a 5% CO_2_ atmosphere. For the experimental trials, the cells were seeded without G418. Cells tested negative for Simian virus 5 and mycoplasma contaminations. Omicron KP.3.1.1 (GISAID accession number: EPI_ISL_19507960) and Omicron XEC (EPI_ISL_19616050) were isolated on air-liquid interface cultures of human bronchial airway epithelial cells and Vero E6/TMPRSS2 cells, respectively. Other virus strains used were isolate BavPat1/984 (variant B.1), Omicron BA.2, Omicron BA.5, Omicron EG.5.1, Omicron JN.1, as described in reference 2. Low-passage stock solutions were generated and titrated on Vero E6/TMPRSS2 cells for all viruses. Virus stocks were sequenced to check for the presence of substitutions introduced during isolation and passaging, and no fixed substitutions or minority variants above 6% frequency were found.

### Plaque reduction neutralization tests

The neutralizing activity of each serum against the different virus strains was determined by plaque reduction neutralization test (PRNT), with small alterations compared to reference 17. In short, Vero E6/TMPRSS2 cells (1.6 × 10^5^ cells/well) were seeded in 24-well plates and incubated for ~24 h. Human sera were serially diluted in OptiPro medium (Gibco) and mixed with medium containing 100 plaque-forming units of the respective virus, incubated at 37°C for 1 h, and then added to the Vero E6/TMPRSS2 cells in duplicate. After a further hour at 37°C, supernatants were discarded, and the cells washed once with phoshpate-buffered saline (PBS) and supplemented with 1.2% Avicel solution in DMEM. After 2 (B.1) or 3 days (all other viruses) at 37°C, the supernatants were removed, and the plates were fixed using a 6% formaldehyde/PBS solution and stained with crystal violet. Plaques were counted for each well or up to two dilutions without plaque reduction, and additional wells were treated as being equal to the seeding dose. If endpoint titers were not reached, titrations using additional serum dilutions were performed and included in the analysis.

### Analysis of neutralization titers

Titers were determined as the dilution where 50% of plaques were neutralized using the “neutcurve” package (version 2.1.0, https://jbloomlab.github.io/neutcurve/) in Python (version 3.12.4, https://www.python.org/), constraining the lower end of the neutralization curve at zero. For the maximal number of plaques for one set of titrations, the average of the number of plaques in the virus control (incubated without serum) or the average of all dilutions with plaque counts above the virus control were used if multiple dilutions had higher plaque counts than the virus control. Geometric mean titers (GMTs) and fold changes were estimated using the gmt and log2diff functions, respectively, in the “titertools” package (version 0.0.0.9003, https://github.com/shwilks/titertools) in R (version 4.4.1, https://www.r-project.org/), as described in reference 3. We used the following parameters: ci_method=“HDI” (highest density interval), ci_level = 0.95, dilution_stepsize = 0, the prior for the mean was a normal distribution with mean of 0 and standard deviation of 100, the prior for the standard deviation was an inverse gamma distribution with a shape parameter of 2 and a scale parameter of 0.75.

## Data Availability

Raw data and code are available upon request. The sequences of SARS-CoV-2 strains are available on GISAID (https://gisaid.org) under the following accession numbers: EPI_ISL_19507960 (KP.3.1.1), EPI_ISL_19616050 (XEC).

## SUPPLEMENTAL MATERIAL

Table S1 mbio.01618-25-s0001.docx Monitoring of anti-SARS-CoV-2 nucleocapsid antibodies.

ASM does not own the copyrights to Supplemental Material that may be linked to, or accessed through, an article. The authors have granted ASM a non-exclusive, world-wide license to publish the Supplemental Material files. Please contact the corresponding author directly for reuse.

## References

[1] Kaku Y, Okumura K, Kawakubo S, Uriu K, Chen L, Kosugi Y, Uwamino Y, Begum MM, Leong S, Ikeda T, Sadamasu K, Asakura H, Nagashima M, Yoshimura K, Genotype to Phenotype Japan (G2P-Japan) Consortium, Ito J, Sato K. 2024. Virological characteristics of the SARS-CoV-2 XEC variant. Lancet Infect Dis 24:e736. doi:10.1016/S1473-3099(24)00731-X 39521009

[2] Jeworowski LM, Mühlemann B, Walper F, Schmidt ML, Jansen J, Krumbholz A, Simon-Lorière E, Jones TC, Corman VM, Drosten C. 2024. Humoral immune escape by current SARS-CoV-2 variants BA.2.86 and JN.1, December 2023. Euro Surveill 29:2300740. doi:10.2807/1560-7917.ES.2024.29.2.2300740 38214083 PMC10785204

[3] Wilks SH, Mühlemann B, Shen X, Türeli S, LeGresley EB, Netzl A, Caniza MA, Chacaltana-Huarcaya JN, Corman VM, Daniell X, et al.. 2023. Mapping SARS-CoV-2 antigenic relationships and serological responses. Science 382:eadj0070. doi:10.1126/science.adj0070 37797027 PMC12145880

[4] Carreño JM, Lerman B, Singh G, Abbad A, Yellin T, Ehrenhaus J, Fried M, Nardulli JR, Kang HM, Mulder LCF, Gleason C, Srivastava K, Simon V, Krammer F, PVI study group. 2025. XBB.1.5 monovalent vaccine induces lasting cross-reactive responses to SARS-CoV-2 variants such as HV.1 and JN.1, as well as SARS-CoV-1, but elicits limited XBB.1.5 specific antibodies. mBio 16:e0360724. doi:10.1128/mbio.03607-24 40042313 PMC11980561

[5] Pušnik J, Monzon-Posadas WO, Osypchuk E, Dubiel AE, Baum M, Fehring P, Büning A, Klant T, Streeck H. 2024. Effect of XBB.1.5-adapted booster vaccination on the imprinting of SARS-CoV-2 immunity. NPJ Vaccines 9:231. doi:10.1038/s41541-024-01023-7 39572559 PMC11582569

[6] Kotaki R, Moriyama S, Oishi S, Onodera T, Adachi Y, Sasaki E, Ishino K, Morikawa M, Takei H, Takahashi H, Takano T, Nishiyama A, Yumoto K, Terahara K, Isogawa M, Matsumura T, Shinkai M, Takahashi Y. 2024. Repeated Omicron exposures redirect SARS-CoV-2-specific memory B cell evolution toward the latest variants. Sci Transl Med 16:eadp9927. doi:10.1126/scitranslmed.adp9927 39167666

[7] Suntronwong N, Kanokudom S, Duangchinda T, Chantima W, Pakchotanon P, Klinfueng S, Puenpa J, Thatsanathorn T, Wanlapakorn N, Poovorawan Y. 2025. Neutralization of omicron subvariants and antigenic cartography following multiple COVID 19 vaccinations and repeated omicron non JN.1 or JN.1 infections. Sci Rep 15:1454. doi:10.1038/s41598-024-84138-0 39789099 PMC11718010

[8] Fossum E, Vikse EL, Robertson AH, Wolf A-S, Rohringer A, Trogstad L, Mjaaland S, Hungnes O, Bragstad K. 2025. Low levels of neutralizing antibodies against SARS‐CoV‐2 KP.3.1.1 and XEC in serum from seniors in May 2024. Influenza Resp Viruses 19. doi:10.1111/irv.70102 PMC12078752 40369880

[9] Wang Q, Guo Y, Mellis IA, Wu M, Mohri H, Gherasim C, Valdez R, Purpura LJ, Yin MT, Gordon A, Ho DD. 2025. Antibody evasiveness of SARS-CoV-2 subvariants KP.3.1.1 and XEC. Cell Rep 44:115543. doi:10.1016/j.celrep.2025.115543 40202847 PMC12014523

[10] Uriu K, Kaku Y, Uwamino Y, Fujiwara H, Saito F, TGtPJ C, Sato K. 2025. Antiviral humoral immunity induced by JN.1 monovalent mRNA vaccines against SARS-CoV-2 omicron subvariants including JN.1, KP.3.1.1, and XEC. Lancet Infect Dis 25:e61. doi:10.1016/S1473-3099(24)00810-7 39672184

[11] Li P, Faraone JN, Hsu CC, Chamblee M, Liu Y, Zheng Y-M, Xu Y, Carlin C, Horowitz JC, Mallampalli RK, Saif LJ, Oltz EM, Jones D, Li J, Gumina RJ, Bednash JS, Xu K, Liu S-L. 2025. Role of glycosylation mutations at the N-terminal domain of SARS-CoV-2 XEC variant in immune evasion, cell-cell fusion, and spike stability. J Virol 99. doi:10.1128/jvi.00242-25 PMC11998534 40135879

[12] Arora P, Happle C, Kempf A, Nehlmeier I, Stankov MV, Dopfer-Jablonka A, Behrens GMN, Pöhlmann S, Hoffmann M. 2024. Impact of JN.1 booster vaccination on neutralisation of SARS-CoV-2 variants KP.3.1.1 and XEC. Lancet Infect Dis 24:e732–e733. doi:10.1016/S1473-3099(24)00688-1 39522531

[13] Liu J, Yu Y, Jian F, Yang S, Song W, Wang P, Yu L, Shao F, Cao Y. 2025. Enhanced immune evasion of SARS-CoV-2 variants KP.3.1.1 and XEC through N-terminal domain mutations. Lancet Infect Dis 25:e6–e7. doi:10.1016/S1473-3099(24)00738-2 39586310

[14] Wang W, Bhushan GL, Paz S, Stauft CB, Selvaraj P, Goguet E, Bishop-Lilly KA, Subramanian R, Vassell R, Lusvarghi S, Cong Y, Agan B, Richard SA, Epsi NJ, Fries A, Fung CK, Conte MA, Holbrook MR, Wang TT, Burgess TH, Mitre E, Pollett SD, Katzelnick LC, Weiss CD. 2024. Antigenic cartography using hamster sera identifies SARS-CoV-2 JN.1 evasion seen in human XBB.1.5 booster sera. bioRxiv. doi:10.1101/2024.04.05.588359

[15] Rössler A, Netzl A, Lasrado N, Chaudhari J, Mühlemann B, Wilks SH, Kimpel J, Smith DJ, Barouch DH. 2025. Nonhuman primate antigenic cartography of SARS-CoV-2. Cell Rep 44:115140. doi:10.1016/j.celrep.2024.115140 39754717 PMC11781863

[16] Jian F, Wang J, Yisimayi A, Song W, Xu Y, Chen X, Niu X, Yang S, Yu Y, Wang P, Sun H, Yu L, Wang J, Wang Y, An R, Wang W, Ma M, Xiao T, Gu Q, Shao F, Wang Y, Shen Z, Jin R, Cao Y. 2025. Evolving antibody response to SARS-CoV-2 antigenic shift from XBB to JN.1. Nature 637:921–929. doi:10.1038/s41586-024-08315-x 39510125 PMC11754117

[17] Wölfel R, Corman VM, Guggemos W, Seilmaier M, Zange S, Müller MA, Niemeyer D, Jones TC, Vollmar P, Rothe C, Hoelscher M, Bleicker T, Brünink S, Schneider J, Ehmann R, Zwirglmaier K, Drosten C, Wendtner C. 2020. Virological assessment of hospitalized patients with COVID-2019. Nature 581:465–469. doi:10.1038/s41586-020-2196-x 32235945

