# Supplementary material for: Humoral immunity to current variants of SARS-CoV-2 in exposed adults, September 2023 to September 2024
Source: mBio. 2025 Sep 12;16(10):e01618-25. doi: 10.1128/mbio.01618-25 (PMC12505990; doi:10.1128/mbio.01618-25)
Supplement: Table S1 — Monitoring of anti-SARS-CoV-2 nucleocapsid antibodies. [file mbio.01618-25-s0001.docx]

**Supplemental material**

**Humoral immunity to current variants of SARS-CoV-2 in exposed adults, September 2023 to September 2024**

Lara M. Jeworowski, Barbara Mühlemann, Felix Walper, Marie L. Schmidt, Jenny Jansen, Andi Krumbholz, Terry C. Jones, Victor M. Corman, Christian Drosten

**Table S1: Monitoring of anti-SARS-CoV-2 nucleocapsid antibodies.** Antibody levels were measured every three months. Each row corresponds to an individual. Colors represent the trend in antibody levels relative to the previous sample. Increases of at least 50% are shown in orange, increases <50% or decreases are shown in grey. A cross indicates that no sample was available for testing. “inf” indicates a reported infection prior to the respective sample. *: Antibody increase marginally below 50% (47%).

|  | **Sep 23** | **winter 23/24** | **spring 24** | **summer 24** | **Sep 24** |
| --- | --- | --- | --- | --- | --- |
| **individuals without infections between Sep 23 and Sep 24** |  |  |  |  |  |
|  |  |  |  |  |  |
|  |  |  |  |  |  |
|  |  |  |  |  |  |
|  |  |  |  |  |  |
|  |  |  |  |  |  |
|  |  |  |  |  |  |
|  |  |  |  |  |  |
|  |  |  |  |  |  |
|  |  |  |  |  |  |
|  |  |  |  |  |  |
|  |  |  |  |  |  |
|  | inf |  |  |  |  |
|  | inf |  |  |  |  |
|  | inf |  |  |  |  |
|  | inf |  |  |  |  |
|  | inf |  |  |  |  |
|  | inf |  |  |  |  |
|  | inf |  |  |  |  |
| **individuals with reported infections (identified by PCR and/or rapid antigen tests) between Sep 23 and Sep 24** |  | inf |  |  |  |
|  |  | inf |  |  |  |
|  |  | inf |  |  |  |
|  |  | inf |  |  |  |
|  |  | inf |  |  |  |
|  |  | inf |  |  |  |
|  |  | inf |  |  |  |
|  |  | inf |  |  |  |
|  |  | inf |  |  |  |
|  |  | inf |  |  |  |
|  |  | inf |  |  |  |
|  |  | inf |  |  |  |
|  |  | inf |  |  |  |
|  |  | inf |  |  |  |
|  |  | inf |  |  |  |
|  |  | inf |  |  |  |
|  |  | inf |  |  |  |
|  |  | inf |  |  |  |
|  |  | inf |  |  |  |
|  |  | inf |  |  | inf |
|  |  | inf |  |  | inf |
|  |  |  | inf |  |  |
|  |  |  | inf |  |  |
|  |  |  | inf |  |  |
|  |  |  | inf* |  |  |
|  |  |  |  |  | inf |
|  |  |  |  |  | inf |
|  |  |  |  |  | inf |
|  |  |  |  |  | inf |
|  |  |  |  |  | inf |
|  |  |  |  |  | inf |
| **individuals with unrecognized infections (identified by antibody testing) between Sep 23 and Sep 24** |  |  |  |  |  |
|  |  |  |  |  |  |
|  |  |  |  |  |  |
|  |  |  |  |  |  |
|  |  |  |  |  |  |
|  |  | inf |  |  |  |
|  |  |  | inf |  |  |
|  |  |  |  |  | inf |
